# Which Came First: Burden of Infectious Disease or Poverty?

**DOI:** 10.1371/journal.pbio.1001457

**Published:** 2012-12-27

**Authors:** Jonathan Chase

**Affiliations:** Freelance Science Writer, Honolulu, Hawaii, United States of America

**Figure:**
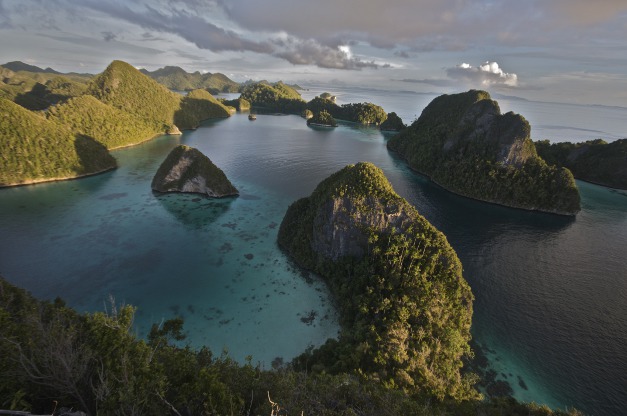
These Raja Ampat Islands are thought to have the greatest marine diversity on earth. By exploring islands like these in the Malay Archipelago (modern day Indonesia), Alfred Russell Wallace (the “father of biogeography") discovered the Wallace Line and crafted a theory of evolution by natural selection. Biogeography can play a similarly important role in the search for underlying drivers of economic development.

A few years ago, I was at an event where the CEO of a coal company was giving the keynote extolling the virtues of “green coal". The crux of his argument was that restrictions on carbon emissions from fossil fuels to generate electricity would in fact be quite detrimental, particularly to the world's poorest people. He backed this up by showing positive correlations between the per capita electricity use in a given country and several indices of the quality of the human condition, which he interpreted to mean that electricity usage causes economic prosperity. Although it was clear that he was twisting cause and effect, my statistical rancor didn't reach its limit until he showed that people from countries with lower per capita electricity use were also more likely to suffer from some of the world's worst infectious diseases and had lowered life expectancies. His concluding slide stated “coal-generated electricity is not only good for the economy, but also good for your health".

Clearly, economics plays a strong role in the burden of infectious diseases. Wealthier countries can invest more in immunizations, control of disease vectors, and treatment following infection, allowing people to live longer, healthier lives, while those in poorer countries are relegated to an often shortened lifetime filled with malady. However, our collective anthropocentric perspective often forgets that we are embedded within a complex ecological web. Nearly two-thirds of the pathogens and parasites that infect humans involve interactions with animals as vectors or alternative hosts. These include some of the worst chronic and emerging diseases we face, including malaria, cholera, and plague. While it's well known that populations with high infectious disease burden are less prosperous, the cause and effect relationships between infectious diseases and prosperity are so intimately intertwined that we have not been able to disentangle whether pathogens drive economies or economies tame pathogens. Said another way, are poor people more likely to get sick, or are sick people more likely to be poor?

A paper published in this issue of *PLOS Biology* by an interdisciplinary team of ecologists and economists led by Matthew Bonds, who has PhDs in both ecology and economics, makes a substantial advance in understanding the interplay between infectious diseases and economic prosperity. It does so by applying a form of structural equation modeling, which allows several different pathways of causality to be examined simultaneously, to disentangle the relative importance of each. The team jointly examined World Bank data on the per capita income of people from 139 countries and the burden of some of the most globally important parasitic and vector-borne diseases, measured as the per capita years of life lost due to mortality and the weighted equivalent of years lost due to morbidity. They also included several other variables known to influence income and infectious disease in a systematic way. For example, economies are known to be strongly influenced by several indicators of governance, including political stability and corruption, while the ecological burden of infectious disease is strongly influenced by latitude (tropical countries have higher burdens than temperate countries).

With the caveat that disentangling cause and effect is always a bit tricky with comparative data, the beauty of the statistical approach employed is that it allowed the investigators to evaluate the relative importance of a potential causal link in the system of equations while controlling for other confounding variables. It also allowed them to evaluate the success of alternative scenarios by which ecology and economics interact. For example, on the economic side, there is a well-known positive relationship between per capita income and latitude; wealthier countries tend to be at higher latitudes, whereas tropical countries tend to be poorer. Economists have typically assumed this to be a historical artifact of colonial expansion from Europe and erroneously discounted the role of pathogens. The current study showed that the latitudinal income gradient most likely results because infectious diseases are more burdensome in the tropics, and that per capita income in many impoverished tropical countries could be doubled simply by reducing the burden of disease to the level found in temperate areas.

On the ecological side, the authors examined an important, but controversial, link between biodiversity and infectious disease burden on an unprecedented scale. On the one hand, biodiversity and infectious disease burden are positively related because increases in infectious disease correlate with increases in biodiversity towards the tropics. On the other hand, there is often a negative relationship between biodiversity and the burden of several important infectious diseases, including Lyme disease, schistosomiasis, West Nile virus, and sin nombre virus. The current study showed that after controlling for the positive covariation between biodiversity and infectious diseases with latitude, a strong negative relationship between biodiversity and disease burden emerged, with direct consequences for per capita income.

While biologists have often borrowed ideas from economics (e.g., game theory), the study by Bonds and colleagues turns the table and shows the utility of ecological ideas for understanding economic systems. Even if the effect of coal-generated electricity on human health remains equivocal, this study provides a compelling case for both the role of infectious diseases in driving economies, and the health benefits of biodiversity conservation, both are of direct relevance to national and global economic prosperity.

**Bonds MH, Dobson AP, Keenan DC (2012) Disease Ecology, Biodiversity, and the Latitudinal Gradient in Income. doi:10.1371/journal.pbio.1001456**

